# P-1120. Successful Management of an Outbreak of Acinetobacter baumannii in an Orthopedic Clinic of a Tertiary Hospital

**DOI:** 10.1093/ofid/ofaf695.1315

**Published:** 2026-01-11

**Authors:** Anna Nikopoulou, Paschalia Skalisti, Sophia Tselenkidou, Natasa Veleni, Soultana Gkiti, Olga Pappa, Menelaos Mountzouris, Kosmas Papadopoulos, Stylianos Skotidakis, Helen Katsifa, Panagiotis Givissis, Damianos Sotiropoulos

**Affiliations:** G. Papanikolaou General Hospital of Thessaloniki, Thessaloniki, Thessaloniki, Greece; G. Papanikolaou General Hospital of Thessaloniki, Greece, Thessaloniki, Thessaloniki, Greece; G. Papanikolaou General Hospital of Thessaloniki, Thessaloniki, Thessaloniki, Greece; G. Papanikolaou General Hospital of Thessaloniki, Thessaloniki, Thessaloniki, Greece; G. Papanikolaou General Hospital of Thessaloniki, Thessaloniki, Thessaloniki, Greece; National Public Health Organization, Athens, Attiki, Greece; G. Papanikolaou General Hospital of Thessaloniki, Thessaloniki, Thessaloniki, Greece; G. Papanikolaou General Hospital of Thessaloniki, Thessaloniki, Thessaloniki, Greece; G. Papanikolaou General Hospital of Thessaloniki, Thessaloniki, Thessaloniki, Greece; G. Papanikolaou General Hospital of Thessaloniki, Thessaloniki, Thessaloniki, Greece; G. Papanikolaou General Hospital of Thessaloniki, Thessaloniki, Thessaloniki, Greece; G. Papanikolaou General Hospital of Thessaloniki, Thessaloniki, Thessaloniki, Greece

## Abstract

**Background:**

Multidrug resistant *A. baumannii* is considered one of the major microbial threats in healthcare settings. The present study emphasizes the importance of timely infection control measures.

**Methods:**

A cluster of 6 cases of *A. baumannii* was detected in the Orthopedic Clinic from 11/6/24 to 11/27/24. The pathogen was isolated from tissue and wound cultures. Surveillance cultures of their contacts were also performed. A total of 31 patients were examined and 31 cultures (rectal and pharyngeal swabs) were obtained, 61 environmental cultures from the Orthopedic Clinic and 32 from the operating theatres. Resistance gene tests were also carried out on 2 of the isolated strains.

**Results:**

*A. baumannii* was isolated in 6 patients (2 males and 4 females). All strains isolated had an extremely drug resistant (XDR) phenotype with sensitivity to colistin and ±tigecycline. Genetic testing of the 2 strains revealed blaNDM, blaOXA-23, blaOXA-51 genes. Upon detection of the initial cases, the transmission route of the pathogen was immediately investigated. Similarities in the hospitalization of patients were sought, and the path of the patients in the hospital was closely examined with regard to the wards, the operating room and the possible stay in the intensive care unit. The Infection prevention and control team implemented measures to suppress the epidemic (Table 1). The epidemiological investigation revealed that the pathogen was most likely transmitted by the hands of the staff, most probably when changing wound dressings. One patient who had previously been hospitalized in the intensive care unit was considered as case zero. Surveillance cultures from contact patients were all negative. Environmental cultures were positive for *Acinetobacter* in the linen storage area, which was cleaned and disinfected with hydrogen peroxide vaporization. Disinfection with hydrogen peroxide vapor was also carried out on other wards of the clinic, in the plaster room and in the operating theaters. Implementation of the measures led to successful control of the outbreak.

**Conclusion:**

Timely implementation of intensive infection control measures, increased adherence to these measures, continued surveillance, and education and training of healthcare workers led to control of the *A. baumannii* outbreak.

**Disclosures:**

All Authors: No reported disclosures

